# Effect of calcium plus Vitamin-D combined with calcitriol in the treatment of patients with Type-2 diabetes and osteoporosis: A retrospective observational analysis

**DOI:** 10.12669/pjms.40.3.8795

**Published:** 2024

**Authors:** Jinxing Wan, Jun Xie, Chaohui Hu, Jiahe Liu, Chunyan Zhu

**Affiliations:** 1 Jin Xing Wan, Department of Endocrinology, Quzhou Affiliated Hospital of Wenzhou Medical University, Quzhou Peoples Hospital Zhejiang Province 100 Minjiang Avenue, Quzhou City, Zhejiang Province 324000, P.R. China; 2 Jun Xie Department of Orthopedics, Quzhou Affiliated Hospital of Wenzhou Medical University, Quzhou Peoples Hospital Zhejiang Province 100 Minjiang Avenue, Quzhou City, Zhejiang Province 324000, P.R. China; 3 Chaohui Hu Department of Endocrinology, Quzhou Affiliated Hospital of Wenzhou Medical University, Quzhou Peoples Hospital Zhejiang Province 100 Minjiang Avenue, Quzhou City, Zhejiang Province 324000, P.R. China; 4 Jiahe Liu Department of Endocrinology, Quzhou Affiliated Hospital of Wenzhou Medical University, Quzhou Peoples Hospital Zhejiang Province 100 Minjiang Avenue, Quzhou City, Zhejiang Province 324000, P.R. China; 5 Chunyan Zhu Department of Endocrinology, Quzhou Affiliated Hospital of Wenzhou Medical University, Quzhou Peoples Hospital Zhejiang Province 100 Minjiang Avenue, Quzhou City, Zhejiang Province 324000, P.R. China

**Keywords:** Calcium plus Vitamin-D, Calcitriol, Type-2 diabetes, Osteoporosis

## Abstract

**Objective:**

To explore the clinical effect of calcium plus Vitamin-D combined with calcitriol in the treatment of patients with type-2 diabetes mellitus (T2DM) patients and osteoporosis.

**Methods:**

In this retrospective observational study clinical records of 90 patients with T2DM combined with osteoporosis, treated in The Quzhou Affiliated Hospital of Wenzhou Medical University from October 2019 to April 2022 were incuded. All patients received basic hypoglycemic treatment. Of 90 patients, 43 received calcium plus Vitamin-D adjuvant therapy (Control-group), and 47 patients received calcium plus Vitamin-D combined with calcitriol adjuvant therapy (Observation-group). Clinical efficacy, adverse reactions, bone metabolism levels, and changes in bone density levels were compared between the two groups.

**Results:**

The clinical efficacy of the treatment was significantly higher in the Observation-group (93.6%) compared to the Control-group (83.7%; *p*<0.05). There was no statistically significant difference in the incidence of adverse reactions between the two groups (*p*>0.05). After treatment, bone metabolism and bone density indicators in both groups improved, and were significantly better in the Observation-group compared to the Control-group (*p*<0.05).

**Conclusions:**

Combination of calcium plus Vitamin-D and calcitriol adjuvant therapy in patients with T2DM and osteoporosis is safe and associated with better treatment efficacy, improved bone metabolism and bone density parameters than calcium plus Vitamin-D treatment alone.

## INTRODUCTION

Type-2 diabetes mellitus (T2DM) and osteoporosis are two common chronic diseases with high prevalence.^1^1DM-induced bone fragility was recently reported as a diabetic complication. This disorder needs to be identified and diagnosed early and adequately to avoid more symptoms and impairments. Bone weight is lowered and the risk of fractures rises in type 1 diabetes mellitus (T1DM Approximately 6.28% of the global population were affected by T2DM, and 10-30% women aged 40 affected by osteoporosis, while up to 10% of men.^2^,^3^ In recent years, studies have showed a certain correlation between T2DM and osteoporosis, and reported that T2DM patients may have abnormal bone metabolism, which is a risk factor for the occurrence of osteoporosis.^4^,^5^ T2DM combined with osteoporosis can increase the complexity and severity of the patient’s condition, affect health, and reduce quality of life.^5^,^6^

The current treatment methods for T2DM combined with osteoporosis mainly include blood glucose control, calcium and Vitamin-D supplementation, and exercise therapy.^5^-^7^ For severe osteoporosis, medication such as bisphosphate can be considered.^7^,^8^ Calcium plus Vitamin-D supplements increase calcium and Vitamin-D3 levels, thus promoting bone formation and increasing bone density.^9^ However, supplementing calcium and Vitamin-D alone cannot promote calcium and phosphorus absorption, which may limit the efficacy of the treatment.^8^ As a Vitamin-D metabolite, calcitriol not only stimulates the intestinal absorption of calcium and phosphorus, but also inhibits the activity of bone resorption cells and preventing bone loss.^10^ Therefore, a combination of calcium plus Vitamin-D and calcitriol therapy may improve the efficacy of the treatment in patients with T2DM and osteoporosis.^8^,^10^,^11^

Data on the efficacy of the combination of calcium plus Vitamin-D and calcitriol for the treatment of T2DM with osteoporosis are scarce. In recent years, our hospital has used calcium plus Vitamin-D combined with calcitriol to treat T2DM patients with osteoporosis. Therefore, the aim of this retrospective analysis was to investigate the efficacy of the combined calcium, Vitamin-D and calcitriol regimen in treating T2DM patients with osteoporosis. Our results may provide reference for healthcare practitioners who are directly involved in treating this group of patients.

## METHODS

In this retrospective observational study, clinical records of T2DM patients with osteoporosis who received treatment in The Quzhou Affiliated Hospital of Wenzhou Medical University from October 2019 to April 2022 were retrospectively reviewed. All patients received basic hypoglycemic treatment. Additionally, 43 patients received calcium plus Vitamin-D adjuvant therapy were set as the Control-group, while 47 patients received calcium plus Vitamin-D combined with calcitriol adjuvant therapy were assigned to the Observation-group.

### Inclusion criteria:

- Patient who were diagnosed with T2DM according to the relevant diagnostic criteria of the Chinese Guidelines for the Prevention and Treatment of T2DM (2020 Edition),^12^ and were diagnosed with osteoporosis based on the relevant diagnostic standards in the “Chinese Guidelines for the Diagnosis and Treatment of Elderly Osteoporosis (2018)” and has passed bone density testing.^13^
- Patient received calcium plus Vitamin-D or calcium plus Vitamin-D combined with calcitriol adjuvant therapy for at least one year of continuous treatment.
- Patients older than 50 years old.

### Exclusion criteria:

- Concomitant endocrine diseases involving thyroid and parathyroid glands.
- Accompanying malignant tumors.
- Severe liver and kidney dysfunction.
- Diagnosis of pathological or hormonal osteoporosis.
- Allergic reaction to the drugs used in the study.
- Bone loss or fracture.
- Incomplete clinical data.

### Ethical Approval:

This study was approved by the Ethics Committee of Biomedical Research Involving Humans of Quzhou People’s Hospital (Approval No.: 2023-043; Date: 2023-05-29).

All patients received basic hypoglycemic treatment. Based on this, patients in the observation-group received calcium plus Vitamin-D combined with calcitriol, and patients in the control-group received calcium plus Vitamin-D. The course of treatment was six months.

### Basic hypoglycemic treatment:

All patients were provided with adequate exercise and dietary guidance, and received metformin sustained-release tablets (Nanjing Yihua Pharmaceutical Co., Ltd.; Specification: 0.5g/tablet; Approval No.: H20040816), twice a day, one tablet orally.

### Calcium plus Vitamin-D regimen:

Patient orally received one daily tablet containing 600mg of calcium/125 international units of Vitamin-D3 (Wyeth Pharmaceutical Co., Ltd.; Approval No.: H10950029), once a day.

### Calcium plus Vitamin-D plus Calcitriol-

In addition to calcium plus Vitamin-D tablet, patient received a capsule containing 0.25ug calcitriol [Zhengda Pharmaceutical (Qingdao) Co., Ltd., Approval No.: H20030491)], once a day, one capsule each time.

### Observation indicators:

***Efficacy:*** Treatment effect was characterized as significant, effective or invalid based on the following criteria: Significant effect - the symptoms of back pain and limb pain have been significantly alleviated, and bone density has significantly increased; Effective - pain symptoms in the back and limbs have been alleviated, and bone density has been improved to a certain extent; Invalid - The symptoms of back and limb pain have not changed, and bone density has not increased or even decreased. Total effective rate = (number of significantly effective cases + number of effective cases)/total number of cases × 100.0%.^12^

### Adverse reactions:

Nausea, vomiting, headache, high blood calcium, and kidney stones.

### Bone metabolism:

Fasting venous blood (3ml) was collected, centrifuged at 4000rpm for eight minutes, and serum was extracted. Levels of procollagen I N-terminal peptide (PINP) and C-terminal telopeptide of collagen type (β-CTX) were measured using electrochemiluminescence method. Parathyroid hormone (PTH), bone alkaline phosphatase (bALP), and 1,25-hydroxyVitamin-D [1,25- (OH) D3] were measured using radioimmunoassay. The reagent kits used were all purchased from Roche Diagnostic Products (Shanghai) Co., Ltd.

### Bone density:

The bone mineral density values of the neck of femur, lumbar spine L2~4 and femoral trochanter were measured with the X-ray bone densitometer (HOLOGIC, model discovery, USA).

### Statistical analysis:

All data analysis was conducted using SPSS 26.0 software (IBM Corp, Armonk, NY, USA). The normality of the data was evaluated using the Shapiro Wilk test. The data of normal distribution were expressed as mean ± standard deviation. The inter-group comparison was conducted by independent sample t test, and the intra-group comparison was conducted by paired t-test. Data of non-normal distribution were expressed as median and interquartile interval. Mann Whitney U test was used for inter-group comparison, and Wilcoxon signed rank test was used for intra- group comparison. The counting data were represented by the number of use cases, and Chi-squared test was used. P<0.05 indicated statistical significance.

## RESULTS

A total of 90 patients (25 males and 65 females) who met the eligibility criteria were included in the study. Age range of the patients was 52-85 years, with an average of 69.2±7.5 years. The course of T2DM was 2-9 years, with an average of 4.9±1.8 years. The body mass index (BMI) was 17.9~28.3 kg/m^2^, with an average of 24.0±2.7 kg/m^2^. There was no statistically significant difference in the baseline data between the two groups (*p*>0.05) (Table-I). Compared with the Control-group, the total efficacy of the Observation-group was significantly higher (*p*<0.05). There was no significant difference in the incidence of adverse reactions between the two groups (*p*>0.05) (Table-II).

**Table-I:** Comparison of baseline data between the two groups.

Group	n	Gender (male/female)	Age (year)	Course of diabetes (year)	BMI (kg/m^2^)
Control-group	43	13/30	63.51±3.58	4(3, 6)	24.5(21.5, 25.7)
Observation-group	47	12/35	64.02±4.12	5(4, 6)	24.5(23.4, 26.5)
*χ^2^*/*t*		0.247	0.618	-1.636	-0.789
*p-Value*		0.619	0.538	0.102	0.430

**Table-II:** Comparison of clinical efficacy and adverse reactions between the two groups.

Group	n	Clinical efficacy				Adverse reactions				

		Significant effect	Effective	Invalid	Total effective rate	Nausea and vomiting	Headache	High blood calcium	Kidney stones	Total occurrence rate
Control-group	43	10 (23.2)	26 (60.5)	7 (16.3)	36 (83.7)	1 (2.3)	1 (2.3)	0 (0)	0 (0)	2 (4.6)
Observation-group	47	23 (48.9)	21 (44.7)	3 (6.4)	44 (93.6)	2 (4.3)	1 (2.1)	1 (2.1)	1 (2.1)	5 (10.6)
*χ^2^*	7.089									1.122
*p-Value*	0.029			0.289						

Before treatment, there was no significant difference in bone metabolism indicators between the two groups of patients (*p*>0.05). After treatment, the PINP, parathyroid hormone, and β-CTX levels in both groups decreased compared to those before treatment, and the Observation-group exhibited significantly lower levels (*p*<0.05). After treatment, the levels of 1,25-(OH) D3 in both groups increased and were markedly higher in the Observation-group compared to the Control-group (*p*<0.05) (Table-III). Levels of bone density indicators before the treatment were comparable in both groups (*p*>0.05). After treatment, bone density levels of neck of femur, lumbar L2~4 and femoral trochanter in both groups were higher than those before treatment. Combined treatment was associated with significantly higher bone density compared to calcium plus Vitamin-D treatment alone (*p*<0.05) (Table-IV).

**Table-III:** Comparison of changes in bone metabolism levels between the two groups.

Group	n	PINP (ng/ml)		PTH (pg/ml)		β―CTX (pg/ml)		1,25-(OH)D3 (ng/ml)	

		Before treatment	After treatment	Before treatment	After treatment	Before treatment	After treatment	Before treatment	After treatment
Control-group	43	74.9±7.4	63 (60, 67)^a^	73 (70, 79)	64 (61,68)^a^	698 (635,798)	592 (529,692)^a^	23.9±2.9	35.3±3.2^a^
Observation-group	47	74.2±6.4	52 (49, 57)^a^	72 (69, 77)	53 (50,58)^a^	725 (625,821)	524 (424,605)^a^	23.1±3.2	42.3±3.4^a^
t		0.492	-5.891	-0.789	-6.211	-0.646	-3.486	1.307	-9.768
p-Value		0.624	<0.001	0.430	<0.001	0.518	<0.001	0.195	<0.001

***Note:*** Compared to before treatment in this group,

a p<0.05.

**Table-IV:** Comparison of bone mineral density changes between the two groups (g/cm²).

Group	n	Neck of femur		Lumbar L2~4		Femoral trochanter	

		Before treatment	After treatment	Before treatment	After treatment	Before treatment	After treatment
Control-group	43	0.6(0.5, 0.8)	0.71±0.20^a^	0.8(0.6, 0.8)	0.9(0.6, 0.9)^a^	0.6(0.5, 0.8)	0.7(0.6, 0.9)^a^
Observation-group	47	0.5(0.4, 0.7)	0.84±0.26^a^	0.7(0.5, 0.9)	0.9(0.7, 1.1)^a^	0.6(0.5, 0.7)	0.8(0.7, 1.0)^a^
t/Z		-1.062	-2.760	-0.726	-2.507	-0.897	-2.422
p-Value		0.288	0.007	0.468	0.012	0.369	0.015

***Note:*** Compared to before treatment in this group,

a p<0.05.

## DISCUSSION

The present study showed that calcium plus Vitamin-D combined with calcitriol is more effective and safer as the adjuvant treatment of T2DM combined with osteoporosis than calcium plus Vitamin-D regimen. Liao et al.^14^ found that the combined treatment using calcium plus Vitamin-D, calcitriol and vitamin K can significantly improve bone density level in male patients with osteoporosis. Avenell et al.^15^ conducted a review of 53 trials and showed that supplementing with Vitamin-D and calcium can prevent osteoporotic fractures in the elderly population without increasing the risk of death. Similarly, our results demonstrate a significant clinical effect of treatment with calcium plus Vitamin-D combined with calcitriol in patients with T2DM complicated by osteoporosis.

This study shows that calcium plus Vitamin-D combined with calcitriol can significantly improve bone metabolism of the patients. T2DM is a persistent high glucose state caused by glucose metabolism disorders. High glucose environment can induce oxidative stress, and lead to the accumulation of glycation end products, and a series of inflammatory reactions in the body. The resulting vascular inflammatory damage and the negative impact on the bone cell formation may lead to abnormal calcium and phosphorus metabolism.^16^,^17^ Studies have found that abnormal calcium and phosphorus metabolism in diabetes patients can affect bone density and bone metabolism.^18^ Ghodsi et al.^19^ pointed out that hyperlipidemia can cause thickening of the microvascular basement membrane, impact vascular blood microcirculation, and the blood supply of bone cells, resulting in the decrease in bone metabolism levels. As a result, a large amount of blood calcium is excreted from the body with urine, ultimately leading to the decrease in bone density. Calcium plus Vitamin-D is a bone metabolism mediator. Oral administration of Calcium plus Vitamin-D can increase intestinal calcium absorption, stabilize the internal bone calcium environment, promote calcium and phosphorus deposition in bone, bone mineralization, and increase bone density.^8^,^20^ Calcitriol, on the other hand, is an active form of Vitamin-D that can promote calcium absorption and increase the bone mass.^10^,^21^

It should be noted that both calcium plus Vitamin-D and calcitriol are drugs that supplement calcium nutrition in the human body.^8^,^20^ The combination of these two drugs may also increase the risk of adverse reactions in patients, such as hypercalcemia and kidney stones.^20^,^21^ Some studies have shown that the dosage of calcitriol used in the treatment of osteoporosis patients can affect the efficacy of the treatment. A dosage of 0.25μg is considered insufficient, while a dosage of 0.5μg can improve the efficacy.^11^,^14^ In our study, a combined regimen used a low (0.25 μg) dose of calcitriol. Nevertheless, the efficacy of the combined treatment was significantly improved compared to calcium plus Vitamin-D treatment alone. Combined treatment, therefore, allows to reduce the dosage of calcitriol, to avoid potential adverse effects and further ensure good medication safety.

Several studies have found that the combination of calcitriol and calcium for the treatment of diabetes with osteoporosis can significantly improve the bone density index of patients without increasing the rate of adverse reactions.^22^ This is consistent with the results of our study. Studies, comparing the effects of calcitriol and calcium carbonate D3 for the treatment of osteoporosis, showed that calcitriol is equally safe but significantly more efficient in improving bone density and bone metabolism in patients with osteoporosis.^23^ This is somewhat different from the combined medication regimen in our study, but can still confirm that the application of calcitriol in the treatment of osteoporosis has certain advantages.

Our current study indicated that calcium plus Vitamin-D combined with calcitriol was significantly associated with improved clinical efficacy in patients with T2DM and osteoporosis. Future studies should further examine the effect of this combined therapy in different populations to improve its clinical application.

### Limitations:

This is a single-center retrospective study with a small sample size. In addition, the follow-up period was only 12 months, and there was no monitoring of patient’s blood glucose indicators. This makes it difficult to evaluate the long-term efficacy and safety of the combination of two drugs.

## CONCLUSION

Combination of calcium plus Vitamin-D and calcitriol adjuvant therapy in patients with T2DM and osteoporosis is safe and results in better treatment efficacy, improved bone metabolism and bone density levels compared to calcium plus Vitamin-D regimen alone.

### Authors’ contributions:

**JW** conceived and designed the study.

**JX, CH, JL and CZ** collected the data and performed the analysis.

**JW** was involved in the writing of the manuscript and is responsible for the integrity of the study.

All authors have read and approved the final manuscript.
